# Systemic increase of AMPA receptors associated with cognitive impairment of long COVID

**DOI:** 10.1093/braincomms/fcaf337

**Published:** 2025-10-01

**Authors:** Yu Fujimoto, Hiroki Abe, Tsuyoshi Eiro, Sakiko Tsugawa, Meiro Tanaka, Mai Hatano, Waki Nakajima, Sadamitsu Ichijo, Tetsu Arisawa, Yuuki Takada, Kimito Kimura, Akane Sano, Koichi Hirahata, Nobuyuki Sasaki, Yuichi Kimura, Takuya Takahashi

**Affiliations:** Department of Physiology, Yokohama City University Graduate School of Medicine, Yokohama 236-0004, Japan; Department of Physiology, Yokohama City University Graduate School of Medicine, Yokohama 236-0004, Japan; Department of Psychiatry, Yokohama City University Graduate School of Medicine, Yokohama 236-0004, Japan; Department of Physiology, Yokohama City University Graduate School of Medicine, Yokohama 236-0004, Japan; Department of Physiology, Yokohama City University Graduate School of Medicine, Yokohama 236-0004, Japan; Department of Physiology, Yokohama City University Graduate School of Medicine, Yokohama 236-0004, Japan; Department of Physiology, Yokohama City University Graduate School of Medicine, Yokohama 236-0004, Japan; Department of Physiology, Yokohama City University Graduate School of Medicine, Yokohama 236-0004, Japan; Radioisotope Research Centre, Yokohama City University Graduate School of Medicine, Yokohama 236-0004, Japan; Department of Physiology, Yokohama City University Graduate School of Medicine, Yokohama 236-0004, Japan; Department of Physiology, Yokohama City University Graduate School of Medicine, Yokohama 236-0004, Japan; Department of Physiology, Yokohama City University Graduate School of Medicine, Yokohama 236-0004, Japan; Hirahata Clinic, Tokyo 150-0002, Japan; Department of Rehabilitation Medicine, St. Marianna University School of Medicine, Kawasaki 216-8511, Japan; Faculty of Informatics, Cyber Informatics Research Institute, Kindai University, Higashi-Osaka 577-8502, Japan; Department of Physiology, Yokohama City University Graduate School of Medicine, Yokohama 236-0004, Japan

**Keywords:** COVID-19, long COVID, cognitive impairment, positron-emission tomography (PET), α-amino-3-hydroxy-5-methyl-4-isoxazolepropionic acid receptor (AMPA receptor)

## Abstract

Long COVID primarily presents with persistent cognitive impairment (Cog-LC), imposing a substantial and lasting global burden. Even after the pandemic, there remains a critical global need for diagnostic and therapeutic strategies targeting Cog-LC. Nevertheless, the underlying neural mechanisms remain poorly understood. Given the central role of synapses in brain function, investigation of synaptic molecular changes may provide vital insights into Cog-LC pathophysiology. In this study, we used [^11^C]K-2 PET to characterize the density of AMPA receptors (AMPARs) on the post-synaptic cell surface, which are crucial synaptic components in brain signalling. Statistical parametrical mapping was used to spatially normalize and apply independent *t*-test for a voxel-based comparison. We selected patients with Cog-LC (*n* = 30) based on Repeatable Battery for the Assessment of Neuropsychological Status assessed persistent cognitive impairment and healthy controls (*n* = 80) with no diagnosed neuropsychiatric disorders. The primary objective was to compare [^11^C]K-2 standardized uptake value ratio with white matter (SUVR_WM_) as a reference region between patients with Cog-LC and healthy controls, and to define the regional extent of differences. The secondary objective was to examine associations between [^11^C]K-2 SUVR_WM_ and plasma concentrations of cytokines or chemokines. As an exploratory objective, we tested whether [^11^C]K-2 PET data could distinguish Cog-LC from healthy controls using a partial least squares based classification algorithm. A voxel-based comparison (*P* < 0.05, *T* > 1.66, one-tailed, false discovery rate control) and a volume of interests analysis (*P* < 0.05, Bonferroni multiple comparison) demonstrated that increased index of AMPAR density in large parts of the brains of patients with Cog-LC compared with that in healthy controls. A voxel-based correlation analysis also showed the brain regions where [^11^C]K-2 SUVR_WM_ correlated positively with plasma TNFSF12 and negatively with plasma CCL2 concentrations. A partial least squares model trained on the index of AMPAR density data demonstrated high diagnostic accuracy, achieving 100% sensitivity and 91.2% specificity. [^11^C]K-2 PET signal represents the index of AMPAR density on the post-synaptic neural cell surface, not on the glial cell surface. A systemic increase in synaptic AMPARs across the brain may drive abnormal information processing in Cog-LC and, through excessive excitatory signalling, pose a risk of excitotoxic neuronal damage. We derived the hypothesis that [^11^C]K-2 PET would be helpful in establishing a diagnostic framework for Cog-LC and that antagonists for cell surface AMPARs, such as perampanel, would be a potential therapeutic target. These hypotheses should be investigated in future large-scale clinical studies.

## Teaser

Fujimoto *et al*. report that the patients with cognitive impairments after SARS-CoV-2 infection showed significant AMPA receptors upregulation across their brains with the [11C]K-2 AMPA receptor PET imaging. These results could offer new strategy for diagnosis and therapeutic approach to overcome cognitive impairment after SARS-CoV-2 infection.

## Graphical Abstract

**Graphical Abstract:**
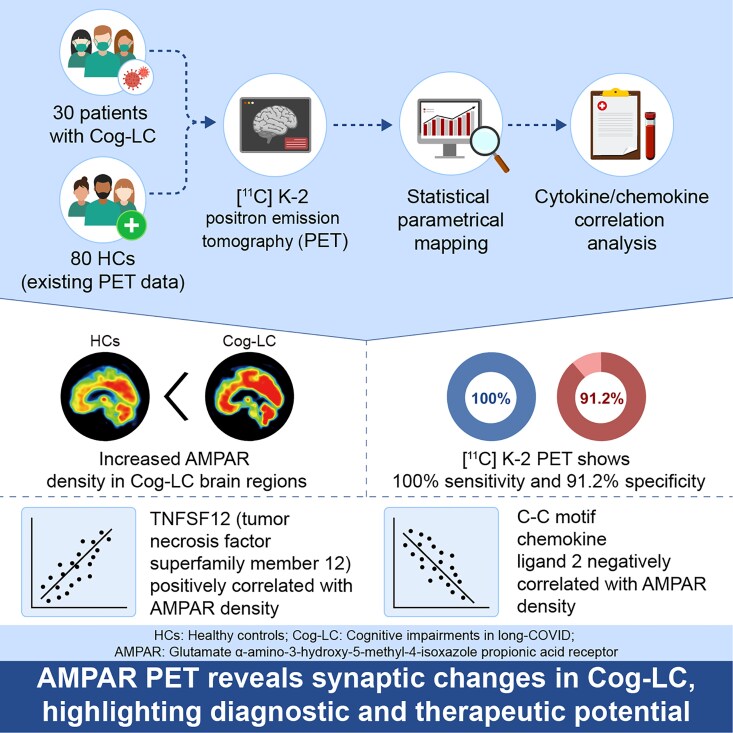

## Introduction

Severe acute respiratory syndrome coronavirus 2 (SARS-CoV-2) infection, also known as COVID-19, may cause an infection-associated chronic condition termed Long COVID.^1^ Long COVID manifests as various symptoms, including cognitive or memory impairments, commonly known as ‘brain fog’.^2^ Cognitive impairments in Long COVID (Cog-LC) have been proposed as a core manifestation of the condition^3^ and occur in over 80% of patients with Long COVID.^4^ Cog-LC is primarily associated with impairments in memory, reasoning and executive functions^5^ and may compel individuals to make occupational changes even months to years after SARS-CoV-2 infection.^6^ Therefore, Cog-LC is considered a major burden not only to individuals’ daily activities but also in the global socioeconomic context, estimated at approximately one trillion dollars resulting from over 400 million global cases of Long COVID.^7^ As such, the accurate diagnosis and effective therapeutic management of Cog-LC represent crucial medical and biological challenges.^8^ Identifying subtypes of Long COVID is crucial, as it is a heterogeneous condition encompassing a range of clinical phenotypes, including respiratory, cardiovascular, neuropsychiatric and cognitive manifestations.^9^ Among these, cognitive impairment (Cog-LC) is particularly challenging to characterize objectively. Currently, the diagnosis of Cog-LC relies primarily on self-reported symptoms, without established biomarkers to confirm or stratify the condition.^7^ This lack of objective diagnostic tools has contributed to under-recognition in clinical practice, delayed intervention and increased patient burden.^10^

Despite these urgent needs, the pathophysiology underlying Cog-LC remains elusive. Investigations with structural and functional magnetic resonance imaging (MRI) in patients with Cog-LC have demonstrated grey matter atrophy and interhemispheric hypoconnectivity in the orbitofrontal cortex and parahippocampus.^11,12^ A study using *N*-isopropyl-*p*-[^123^I]-iodoamphetamine single-photon emission computed tomography identified changes in blood flow primarily in the occipital lobes, which could be ameliorated with repetitive transcranial magnetic stimulation, leading to improvement in memory tests.^13^ These findings are significant; however, they do not provide a sufficient biological basis for diagnosing or treating patients with Cog-LC because the crucial molecules regulating the function of the neurons are not being targeted. Furthermore, alterations in cerebral blood flow do not necessarily reflect primary changes in neuronal activity, but rather may represent secondary or compensatory hemodynamic responses, limiting their utility in elucidating the core molecular pathophysiology of Cog-LC. Neural functions are directly linked to the neuronal synapses, which are fundamental structural units in the brain. Among various synapses, glutamatergic synapses play essential roles in neuronal functions.^14^ Glutamate α-amino-3-hydroxy-5-methyl-4-isoxazole propionic acid receptor (AMPAR) is a principal mediator of glutamatergic synaptic functions and is crucial for learning and memory, the establishment of functional neuronal systems during development, and functional recovery after brain injury.^15-19^ AMPAR abnormalities are implicated in brain pathophysiology.^20^

The investigation of AMPAR in Cog-LC is supported by a growing body of evidence, providing a rationale for its relevance. ^18^Fluorodeoxyglucose (^18^F-FDG) positron emission tomography (PET) tracer studies in patients with Cog-LC have revealed the hypometabolism in the brain areas such as the limbic system, brainstem and cerebellum.^21-24^ Recent animal studies indicate that ^18^F-FDG-PET signals in the brain are largely attributable to astrocytic glucose metabolism.^25^ These findings suggest that ^18^F-FDG-PET hypo signals in Cog-LC may reflect astrocytic dysfunction. Because astrocytes are also essential for synaptic glutamate clearance and recycling, it is plausible that impaired astrocyte mediated glutamate homeostasis contributes to cognitive deficits. However, whether the astrocytic dysfunction could lead pathological changes of glutamate AMPARs on the neural cell surface has not yet been elucidated in patients with Cog-LC. Another previous report has demonstrated that elevated translocator protein total distribution volume (TSPO V_T_) using [¹⁸F]FEPPA PET in patients with depressive and/or cognitive symptoms following COVID-19, indicating that microgliosis and astrocytic gliosis may persist after SARS-CoV-2 infection.^26^ Gliosis has been interpreted as a key neuroinflammatory process, typically resulting from microglial overactivation driven by elevated proinflammatory cytokines. Among these, tumour necrosis factor-alpha (TNF-α) has been shown to directly increase the synaptic trafficking and surface expression of AMPARs.^27^ Given that sustained cytokine elevation is thought to underlie gliosis in Long COVID, it is plausible that AMPARs trafficking to post synaptic membrane may also be elevated by inflammatory factors in patients with Cog-LC. However, no molecular imaging studies to date have directly visualized synaptic AMPARs alterations in patients with Cog-LC. This gap has limited our ability to validate synaptic mechanisms in living human brain and to translate molecular hypotheses into therapeutic strategy.

A technique to monitor AMPAR in the living human brain has long been in demand to reveal the role of this molecule in neuropsychiatric diseases. We recently developed a PET tracer for AMPAR, [^11^C]K-2, which visualizes and quantifies AMPAR density in the living human brain.^28,29^ [^11^C]K-2 demonstrated favourable reversible kinetics, making it suitable for quantifying AMPARs density using PET. We previously demonstrated that the standardized uptake value ratio (SUVR) using the white matter (WM) as a reference region reflects the index of absolute AMPAR density and exhibits positive correlations with the local biochemical amount of AMPARs in surgically resected brain tissues from patients with mesial temporal lobe epilepsy.^28^ After the intravenous administration, [^11^C]K-2 is rapidly metabolized to [^11^C]K-2_OH_, which constitutes the image signals.^30^ In vitro assays indicate that [^11^C]K-2_OH_ crosses the blood–brain barrier but does not penetrate cell membranes.^30^ Thus, [^11^C]K-2 PET depicts the AMPARs on the neural cell surface. Recent studies have demonstrated that AMPARs exist as tetramers on the postsynaptic membrane, whereas monomeric forms are present on the extrasynaptic cell membrane.^31^ The K-2 precursor selectively binds to AMPAR multimers but not to monomers,^32^ indicating that [¹¹C]K-2 PET specifically visualizes AMPARs localized on the postsynaptic membrane.^30^ Recent studies involving [^11^C]K-2 in patients with epilepsy or psychiatric disorders have demonstrated the pathophysiology of these conditions.^33-35^ Animal models of neuropsychiatric diseases, particularly those focusing on cognitive impairments, are usually challenging to interpret for humans owing to their lack of pathophysiology in living humans.^36^ Animal models of Cog-LC have been reported;^37^ however, they are not based on pathophysiological information derived from the living human brain. Therefore, the challenges in using Cog-LC animal models as a basis for diagnostics or drug development persist. In this study, we aimed to investigate AMPAR density in patients with Cog-LC using [¹¹C]K-2 PET imaging. The primary objective of the study was to compare SUVRs using WM as a reference (SUVR_WM_) between patients with Cog-LC and healthy controls (HCs), and to determine the regional distribution of any differences. The secondary objective was to examine the relationship between SUVR_WM_ and plasma levels of cytokines and chemokines. As an exploratory aim, we also tested whether [¹¹C]K-2 PET data could differentiate patients with Cog-LC from HCs using a partial least squares (PLS)-based classification approach.

## Materials and methods

### Ethical statement

This study investigated patients with Cog-LC using [^11^C]K-2 PET imaging and was approved by the Yokohama City University Certified Institutional Review Board (CRB23-004). This trial was conducted at Yokohama City University Hospital between 26 October 2023 and 6 September 2024 following the Japanese Clinical Trials Act. All participants provided written informed consent after receiving detailed information regarding the research protocol.

### Participants

Patients aged 20–59 years with subjective cognitive impairment as sequelae of SARS-CoV-2 infection and no previous history of neuropsychiatric disorders were recruited. The inclusion criteria were as follows: (i) male or female, aged 20–59 years at the time of consent; (ii) confirmed SARS-CoV-2 infection through clinical symptoms (e.g. fever and upper respiratory symptoms) diagnosed by a physician or positive polymerase chain reaction (PCR) or antigen test at a medical institution; (iii) persistent cognitive sequelae lasting for at least 2 months^38^ with ongoing symptoms affecting work, study, or daily life at the time of consent; (iv) Repeatable Battery for the Assessment of Neuropsychological Status (RBANS) score^39^ below 85 after age adjustment (mean 100, SD 15) or a subscale score of ≤−1 SD; and (v) deemed competent to provide consent based on the MacArthur Competence Assessment Tool (MacCAT) evaluation, with written consent obtained from the participants or their legal representatives. The study cohort did not include individuals with a prior history of medical or neurological conditions associated with cognitive impairment. Screening for a history of neuropsychiatric disorders was conducted through clinical interviews performed by both a board-certified psychiatrist and a neurologist. In addition, the Autism Spectrum Quotient (AQ) and Part III of the MDS-sponsored revision of the Unified Parkinson’s Disease Rating Scale (MDS-UPDRS Part III) were administered as supportive assessments.

### Assessment for cognitive impairment and depressive symptoms

RBANS is a standard cognitive screening instrument. It comprises 12 subscales across five domains (Immediate Memory, Visuospatial-Constructional, Language, Attention and Delayed Memory).^40^ We performed the RBANS Japanese version on each participant and obtained 12 subscale and age-based standardized total scores. As previous studies suggested that memory, attention, executive functions were mainly impaired in Cog-LC,^5^ we paid close attention to immediate/delayed memory score and attention score. In addition to RBANS, the enrolled participants underwent Letter Number Sequencing (LNS), Stroop Neuropsychological Screening Test (SNST), Trail Making Test (TMT), and Mini-Mental State Examination (MMSE) to characterize their cognitive capacities. Depressive symptoms were measured using the Montgomery–Åsberg Depression Rating Scale (MADRS) and Hamilton Depression Rating Scale (HAM-D).

### Brain imaging

[^11^C]K-2 PET scanning using Cartesion (Canon Medical, Gunma, 324-8550, Japan) or Celesteion PCA-9000A/2A (Canon Medical, Gunma, 324-8550, Japan) and T1-weighted magnetic resonance imaging using GE DISCOVERY MR750 (General Electric Medical Systems) for patients with Cog-LC were performed at Yokohama City University Hospital. [^11^C]K-2 PET scanning on computed tomography (CT) was performed with 370 MBq ± 10% [^11^C]K-2 bolus venous injection. Summed [^11^C]K-2 PET images were constructed using data collected 30–50 min after venous injection. These images were then converted into SUVR_WM_ images. Subsequently, we aligned the SUVR_WM_ and T1WI images using SPM 12.

### Logan graphical analysis

Logan graphical analysis (LGA) with reference tissue was applied to compute non-displaceable binding potential (*BP*_ND_).^41,42^  *BP*_ND_ is a quantitative unitless index of receptor density that is defined as the ratio of the amount of available receptor sites to the disassociation rate between an administered radiopharmaceutical and its specific binding sites.^43^ LGA with reference tissue is applicable to any compartment model, such as the 1-tissue-2- or 2-tissue-3-compartment model, provided the model can be described by Eq. (1) of Ref.^41^ If the kinetics of the administered radioligand exhibit reversible behaviour, *BP*_ND_ can be calculated using LGA. The tTAC in the reference region, a WM region, was given to the LGA. The beginning of the linear regression, t*, is 20 min after administration.^28^ In all but two cases, five data points from the regression were included. We excluded these two cases owing to the limited PET imaging time (30–50 min post-administration) due to headaches, which was deemed insufficient for obtaining statistically reliable estimates.

### Statistical analysis

The [^11^C]K-2 PET images of HCs were obtained from a previous study (jRCTs031200083), which was predefined in the present Cog-LC study protocol. In the analysis in primary objective, voxel-based comparison of SUVR_WM_ between HCs and patients with Cog-LC was performed using a two-sample *t*-test implemented in Statical Parametric Mapping (SPM) 12. We conducted the correction of multiple comparison in voxel-based with false discovery rate correction (FDRc). In a comparison of SUVR_WM_ in each Hammers’ volume of interests (VOIs) calculated by PNEURO tool (see Supplementary Methods 2), we conducted multiple comparison *t*-tests with Bonferroni correction using GraphPad Prism 10 (Graph Pad Software, Massachusetts, USA).

In the analysis in secondary objectives, the correlation between SUVR_WM_ and the RBANS Scores or plasma concentrations of cytokines or chemokines was determined using a multiple regression design implemented in SPM 12.

In the analysis in exploratory objective, the PLS^44^ model used SUVR_WM_ as the explanatory variable and included response variables such as disease status (0 for HCs, 1 for patients), age and sex. The model was trained on all participants except those comprising the test pair using a leave-one-pair-out cross-validation approach, which was then applied to the test pair to generate predicted diagnostic values. For each participant, values closer to 0 indicated a stronger resemblance to HCs. In contrast, values approaching 1 suggested a higher likelihood of Cog-LCs (Supplementary Fig. 1).

In voxel-based analysis in primary and secondary objectives, statistical significance was set at *P* < 0.05 (uncorrected at peak level), and the FDR was corrected at *P* < 0.05 (cluster-level inference) to account for multiple comparisons across all voxels within the mask. We used an explicit mask before conducting statistical analyses to minimize inter-subject variability and to remove noise from non-brain regions. This mask encompassed areas with a minimum 10% probability of grey matter presence, as defined in SPM 12.

Unpaired *t*-test and the Fisher exact test were used to analyse patient characteristics. Plots were created using GraphPad Prism 10.

## Results

### Participants characteristics and study structure

**Figure 1:**
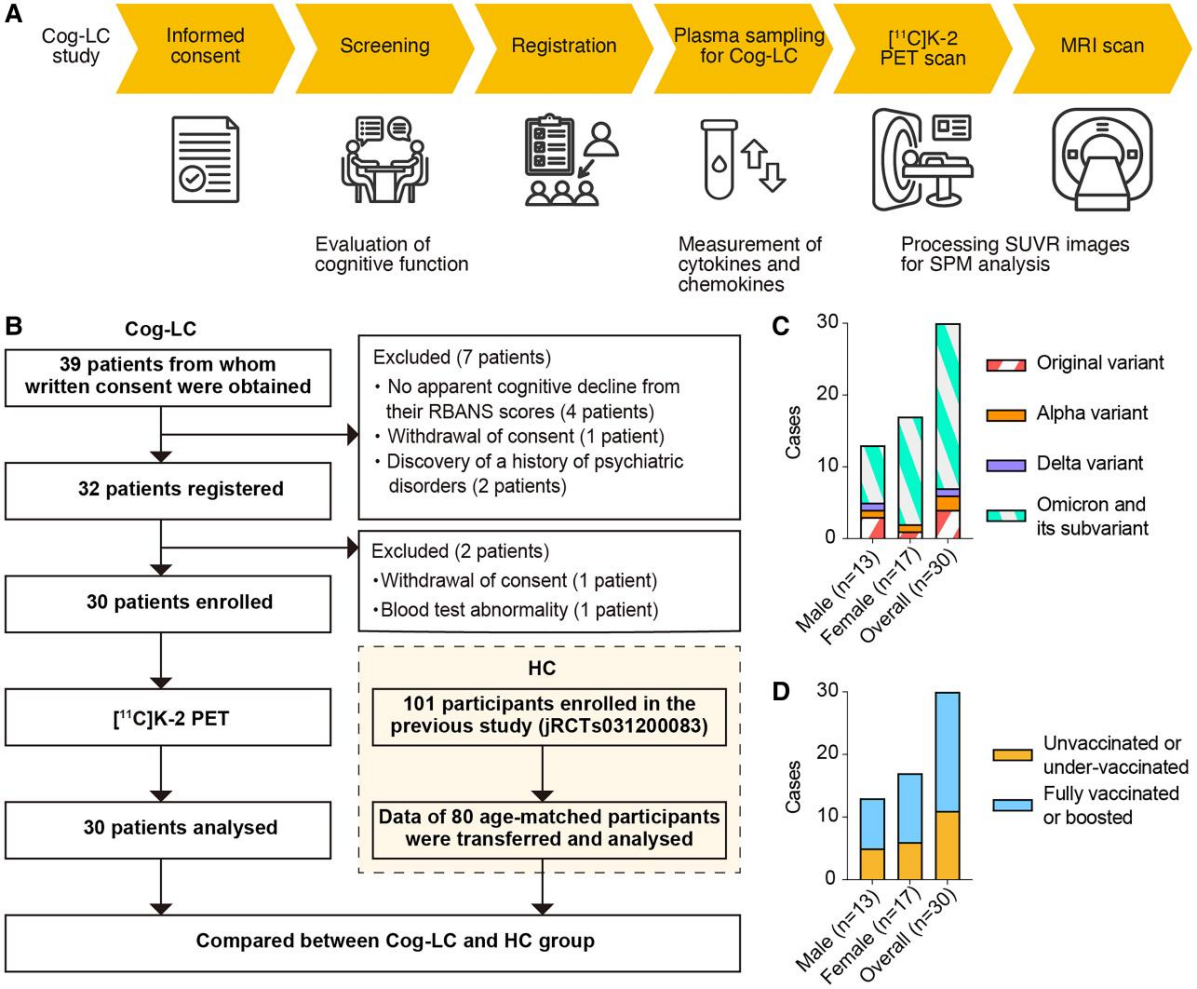
**Study profile.** (**A**) Flowchart of the timeline of the participants. (**B**) CONSORT diagram. (**C**) Infecting variants for each patient, inferred from the date of infection and genomic surveillance data from the National Institute of Infectious Diseases in Japan. (**D**) Number of cases with insufficient or sufficient vaccination before infection. *Abbreviations:* SUVR, standardized uptake value ratio; SPM, statistical parametric mapping; RBANS, repeatable battery for the assessment of neuropsychological status; jRCTs, Japan Registry of Clinical Trials.

In this study, 30 enrolled participants with Cog-LC (jRCTs031230306) underwent plasma sampling, [^11^C]K-2 PET and MRI (Fig. 1A). The present Cog-LC study also utilized data from a previous study that evaluated AMPAR density in healthy individuals using [^11^C]K-2 (jRCTs031200083) (Fig. 1B), and [^11^C]K-2 PET images of 80 healthy age-matched individuals were extracted and used as controls (HCs) (Fig. 1B). The study cohort did not include individuals with a prior history of medical or neurological conditions associated with cognitive impairment (Supplementary Table 1). The SARS-CoV-2 strain variants in participants with Cog-LC comprised omicrons and their subvariants (77.7%), as well as original (13.3%), alpha (6.7%) and delta (3.3%) variants, as deduced based on genomic surveillance data from the National Institute of Infectious Diseases in Japan (Fig. 1C). Among the 30 registered patients with Cog-LC, 11 (36.7%) were unvaccinated or under-vaccinated, and 19 (63.3%) were fully vaccinated or boosted (Fig. 1D).^9^ The patients underwent [^11^C]K-2 PET scanning 22.3 ± 12.4 months (average ± standard deviation [SD], ranging from 5 to 52 months) after COVID-19 onset. In our cohort, we cannot provide the precise information about the duration between onset of cognitive impairment and episode of acute COVID-19. Compared with the HCs, the 30 participants with Cog-LC showed significantly lower total RBANS, subscale (particularly in coding and figure recall), MMSE and LNS scores but comparable SNST and TMT scores (Table 1). The MADRS (9.9 ± 5.9) and HAM-D Scale (8.2 ± 4.7) scores in the Cog-LC group primarily reflected symptoms of reduced sleep and concentration challenges and did not meet the psychiatric diagnostic criteria defined by the Diagnostic and Statistical Manual of Mental Disorders, Fifth Edition (DSM-5) or the International Statistical Classification of Diseases and Related Health Problems 10th Revision (ICD-10). The MDS-UPDRS Part III scores (2.8 ± 5.0) indicated slight difficulties in skilled movement; however, none of the participants met the diagnostic criteria for movement disorders. Therefore, these evaluations indicated that the cohort in the present study exhibited subjective and objective cognitive impairments without diagnosable mental and movement disorders.

**Table 1:** Demographic and clinical characteristics of patients with cognitive impairments in long COVID (Cog-LC) and HCs

	Cog-LC (*n* = 30)	HC (*n* = 80)
Sex—no. (%)		
Male	13 (43.3)	41 (51)
Female	17 (56.7)	39 (49)
Age—years		
Mean ± SD	41.3 ± 9.0	39.6 ± 11.7
Median (IQR)	43.0 (35.8–46.3)	40.0 (28.3–49.8)
Range	23.0–57.0	20.0–57.0
BMI—kg/m^2^		
Mean ± SD	24.0 ± 4.2	22.7 ± 3.8
Median (IQR)	24.0 (20.8–26.1)	22.5 (19.9–24.4)
Range	15.7–36.8	15.8–34.6
Educational background—no. (%)		
Non-higher education	0 (0.0)	2 (2.5)
High-school dropout	0 (0.0)	0 (0.0)
High-school graduate	3 (10.0)	13 (16.3)
College non-completer or associate degree	8 (26.7)	29 (36.3)
Bachelor	15 (50.0)	31 (38.8)
Master or more	4 (13.3)	5 (6.3)
RBANS total score****		
Mean ± SD	67.9 ± 20.0	98.7 ± 14.6
Median (IQR)	71 (56.8–81.0)	100 (86.0–110.0)
Range	2–97	68–133
RBANS subscales (Mean ± SD)		
List learning***	26.8 ± 4.6	30.2 ± 4.1
Story Memory	16.9 ± 4.7	18.8 ± 3.6
Figure copy**	15.3 ± 2.4	18.1 ± 2.1
Line orientation	18.0 ± 2.4	18.5 ± 1.9
Picture naming	9.0 ± 0.9	9.8 ± 0.9
Semantic fluency*	16.0 ± 3.2	18.4 ± 4.0
Digit span	10.3 ± 2.1	12.5 ± 2.5
Coding****	48.3 ± 11.1	61.4 ± 8.3
List recall	5.2 ± 2.2	7.2 ± 2.2
List recognition	19.0 ± 1.5	19.5 ± 0.8
Story recall	9.3 ± 3.1	10.4 ± 1.9
Figure recall****	10.4 ± 3.7	14.5 ± 3.7
Other cognitive scales (Mean ± SD)		
MMSE (score)***	28.0 ± 2.3	29.4 ± 0.8
LNS (score)*	11.8 ± 2.1	13.2 ± 2.6
SNST (Part 3-part 1, s)	6.6 ± 5.4	5.0 ± 3.8
TMT (Part B-part A, s)	7.2 ± 36.1	8.8 ± 24.1
Mood scale (Mean ± SD)		
MADRS****	9.9 ± 5.9	0.1 ± 0.5
HAM-D (21 items)****	8.2 ± 4.7	0.3 ± 0.8

Two Cog-LC patients could not accomplish LNS. SD, standard deviation; IQR, median with interquartile range, * *P* < 0.05, ** *P* < 0.01, *** *P* < 0.001, **** *P* < 0.0001.

### Kinetics of [^11^C]K-2 in patients with Cog-LC

Tissue time activity curves (tTACs) revealed regional heterogeneity in [^11^C]K-2 uptake among multiple brain regions in the cortex, putamen and cerebellum of patients with Cog-LC (Supplementary Fig. 2A). The values of *BP*_ND_ were obtained from LGA with reference tissue. We have previously demonstrated that the total distribution volume, which is a surrogate marker of AMPAR density, was lowest in WM among various brain regions. Additionally, we confirmed by biochemical assay that AMPA receptor expression in WM is negligible,^28^ supporting its use as a reference region. LGA using WM as a reference revealed linearity, demonstrating the reversible binding kinetics of [^11^C]K-2 in these patients (Supplementary Fig. 2B), as previously shown in healthy participants, patients with epilepsy, and those with psychiatric disorders.^28,34,35^ Based on LGA with reference tissue, we calculated *BP*_ND_ in each brain region. To evaluate whether SUVR_WM_ serves as a surrogate for *BP*_ND_, we performed a regression analysis between *BP*_ND_ and SUVR_WM_-1. We found that they exhibited a linear relationship (Supplementary Fig. 2C).^28,34,35^ This regression analysis indicated that SUVR_WM_ could be an appropriate surrogate outcome for measuring AMPAR density in patients with Cog-LC.

### [^11^C]K-2 SUVR_WM_ elevations in Cog-LC

**Figure 2:**
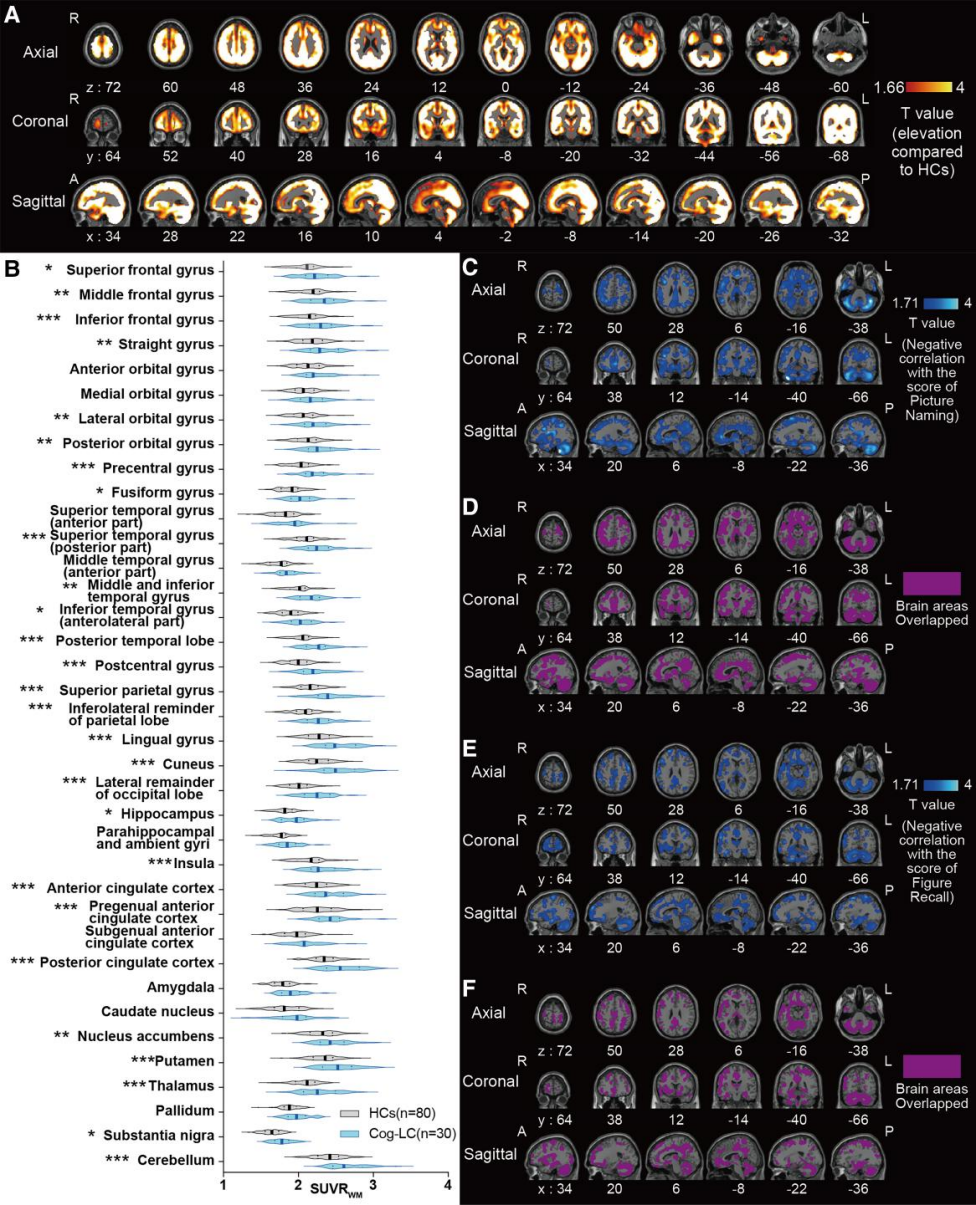
**AMPAR distribution pattern in cognitive impairments in long COVID (Cog-LC).** (**A**) Elevations in [^11^C]K-2 SUVR_WM_ in patients with Cog-LC (*n* = 30) compared to HCs (*n* = 80) (*P* < 0.05, *T* > 1.66, one-tailed, FDRc). (**B**) Multiple comparisons across Hammers’ VOIs between HCs (*n* = 80) and Cog-LC (*n* = 30). Bold line and dashed line of each plot represents mean and quartiles, respectively. *P < 0.05, **P < 0.01, ***P < 0.001 (Bonferroni multiple comparison test after two-way ANOVA analysis). (**C**) Brain regions showing a negative correlation between [^11^C]K-2 SUVR_WM_ and picture-naming scores of the RBANS in Cog-LC (*n* = 30) (*P* < 0.05, *T* > 1.71, one-tailed, FDRc). (**D**) Overlapping brain regions between the clusters in **A** and **C**. (**E**) Brain regions showing a negative correlation between [^11^C]K-2 SUVR_WM_ and figure recall scores of the RBANS in Cog-LC (*n* = 30) (*P* < 0.05, *T* > 1.71, one-tailed, FDRc). (**F**) Overlapping brain regions between the clusters in **A** and **E**. *Abbreviations*: A, anterior; P, posterior; R, right; L, left; FDRc, false discovery rate correction.

We compared the [^11^C]K-2 SUVR_WM_ of patients with Cog-LC to that of HCs. Voxel-wise analysis using SPM indicated a statistically significant increase in the SUVR_WM_ of patients with Cog-LC compared to that of HCs across the brain (Fig. 2A), indicating increased AMPAR density in patients with Cog-LC. Similarly, multiple comparisons across Hammers’ VOIs^45^ showed significantly increased SUVR_WM_ in patients with Cog-LC compared with HCs in multiple VOIs (Fig. 2B). In addition, we noted brain regions where SUVR_WM_ and the scores of the Picture naming and Figure Recall, which are RBANS subcategories, were negatively correlated (Fig. 2C and E), most of which were identified as areas where AMPAR density was elevated in patients with Cog-LC compared with those in HCs (Fig. 2D and F).

### Correlation between plasma cytokines-chemokines and [^11^C]K-2 SUVR_WM_

**Figure 3:**
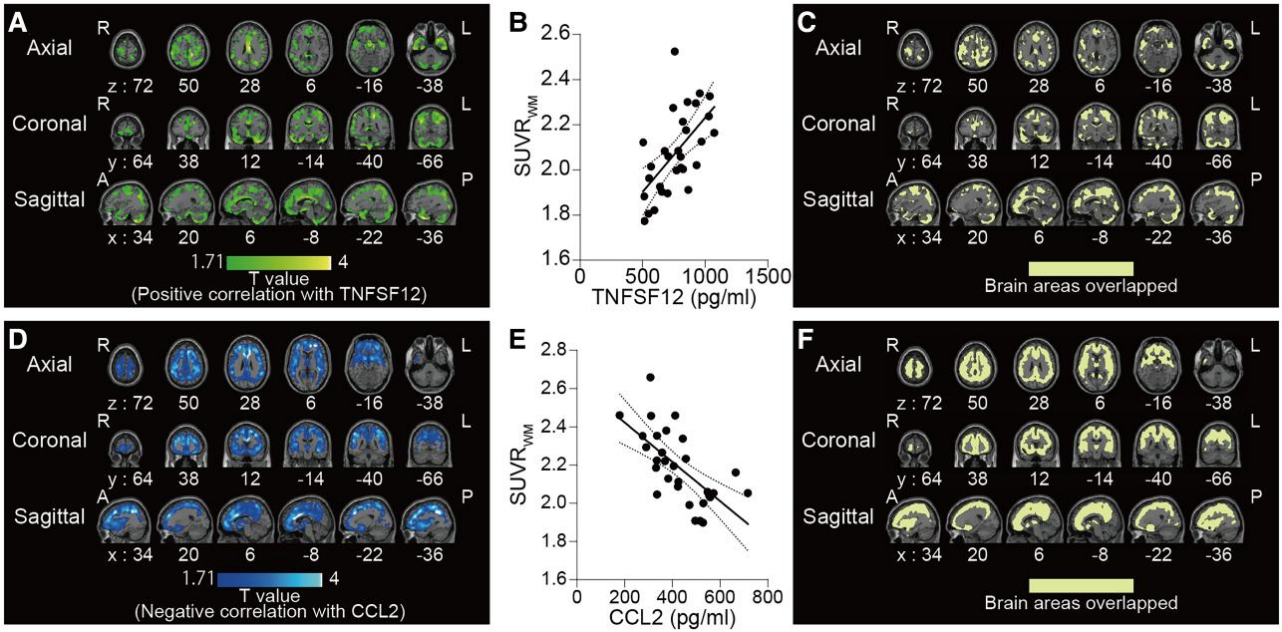
**Correlation between elevated AMPAR density and plasma protein levels.** (**A**) Brain regions showing a significant positive correlation between [^11^C]K-2 SUVR_WM_ and plasma TNFSF12 concentration (*n* = 30, multiple regression analysis using age and sex as covariates, *P* < 0.05, *T* > 1.71, one-tailed, FDRc). (**B**) Correlation between average SUVR_WM_ in a significant cluster and plasma TNFSF12 concentrations (correlation coefficient = 0.6051, *P < 0.001). Each dot represents SUVR_WM_ of the brain region shown in Fig. 3A and plasma TNFSF12 concentration of each participant. (**C**) Overlapping brain regions between the clusters in Figs 2A and 4A. (**D**) Brain regions showing a significant negative correlation between [^11^C]K-2 SUVR_WM_ and plasma CCL2 concentration in Cog-LC (*n* = 30) (*n* = 30, multiple regression analysis using age and sex as covariates, *P* < 0.05, *T* > 1.71, one-tailed, FDRc). (**E**) Correlation between average SUVR_WM_ in a significant cluster and plasma CCL2 concentrations (two-tailed Pearson correlation analysis: correlation coefficient = −0.6583, *P < 0.001). Each dot represents SUVR_WM_ of the brain region shown in **D** and plasma CCL2 concentration of each participant. (**F**) Overlapping brain regions between clusters in Figs 2A and 4D. In **B** and **E**, solid lines represent the regression, and dashed lines represent the 95% CI. *Abbreviations*: TNFSF12, tumour necrosis factor superfamily 12; CCL2, C-C motif chemokine ligand 2; FDRc, false discovery rate correction; A, anterior; P, posterior; R, right; L, left.

Furthermore, we evaluated the correlation between AMPAR density and plasma immunological signatures. Correlations between voxel-wise [^11^C]K-2 SUVR_WM_ and plasma concentrations of cytokines or chemokines in patients with Cog-LC were analysed using SPM. SPM analyses identified brain regions where the plasma concentrations of seven factors, TNFSF12, OLR1, TGF-α, IL-7, TNFSF10, HGF and MMP-12, were positively correlated with voxel-wise [^11^C]K-2 SUVR_WM_ (Fig. 3A and B, Supplementary Fig. 3A–L), and the plasma concentrations of CCL2 and CXCL9 were negatively correlated with voxel-wise [^11^C]K-2 SUVR_WM_ (Fig. 3D and E, Supplementary Fig. 3M and N). Two of the above nine factors, TNFSF12 and CCL2, exhibited a strong significant correlation, defined here as having an absolute Pearson *r* 95% confidence interval (|*r*|) > 0.3, suggesting that they are significantly associated with the upregulation of AMPAR expression in patients with Cog-LC. A strong positive correlation (95% CI of Pearson *r*: 0.31–0.79) was identified between the plasma TNFSF12 levels and AMPAR density in extensive brain areas (Fig. 3A and B). Similarly, these regions substantially overlapped with areas exhibiting elevated [^11^C]K-2 SUVR_WM_ in patients with Cog-LC, relative to those in HCs (Fig. 3C). We observed a significant and robust negative correlation (95% CI of Pearson’s *r*: −0.82 to −0.39) between plasma CCL2 levels and the density of AMPAR across widespread brain regions (Fig. 3D and E). Notably, these regions overlapped primarily with those showing increased [^11^C]K-2 SUVR_WM_ in patients with Cog-LC compared to those in HCs (Fig. 3F).

### PLS analysis of SUVR_WM_ to distinguish HCs and Cog-LC

**Figure 4:**
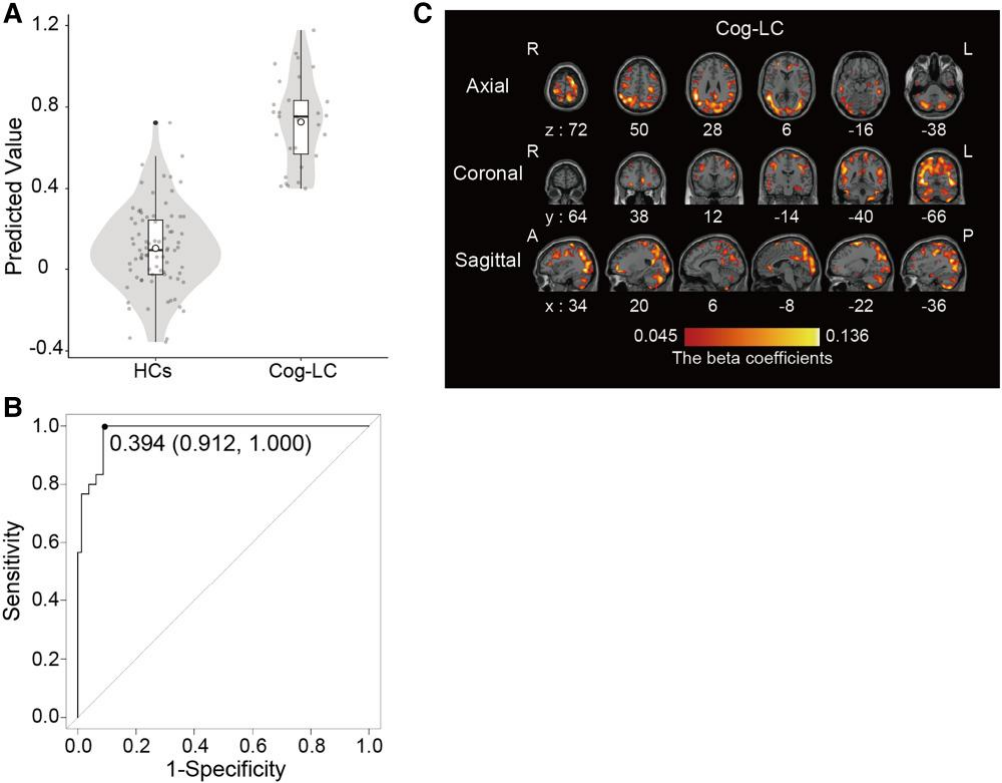
**Diagnostic performance of PLS algorithm.** (**A**) Predicted values from the PLS models are displayed using a violin plot and box plot. Each grey dot represents each participant. The means of the predicted values are represented by circular points. (**B**) Receiver operating characteristic curve (solid line) illustrates the diagnostic performance of the PLS algorithm in distinguishing patients with Cog-LCs from HCs. The area under the curve was 0.980 (95% confidence interval [CI]: 0.960–0.999). The optimal cutoff was 0.394, providing a sensitivity of 100.0% for identifying patients (29 of 30), specificity of 91.25% (73 of 80), positive predictive value of 81.08% (30 of 37), and negative predictive value of 100.0% (73 of 73). (**C**) The beta coefficients from a representative partial least squares model, which are associated with disease status, are shown in the visualization. A higher beta coefficient indicates that the predicted value increases as the SUVR value of a corresponding voxel increases. As a higher predicted value suggests a stronger likelihood of Cog-LCs diagnosis, these coefficients highlight the relationship between specific voxel SUVR values and the probability of distinguishing Cog-LCs from HCs. *Abbreviations:* A, anterior; P, posterior; R, right; L, left; PLS, partial least squares; SUVR, standardized uptake value.

We utilized PLS analysis to illustrate the distribution of the predicted values between participants with Cog-LC and HCs, a scatter plot was constructed (Fig. 4A). Receiver operating characteristic analysis of these predicted values yielded an area under the curve of 0.980 (95% confidence interval [CI]: 0.960–0.999) (Fig. 4B). The optimal threshold, determined using the Youden Index, was 0.394, resulting in a sensitivity of 100%, specificity of 91.25%, positive predictive value of 85.08%, and negative predictive value of 100% (Fig. 4B). The beta coefficients, which are associated with the disease status, were also visualized for the representative PLS model (Fig. 4C). A high beta coefficient indicates that, as the SUVR_WM_ value of a voxel increases, the corresponding predicted value also increases. Because a high predicted value suggests a strong likelihood of Cog-LC diagnosis, these coefficients highlight the relationship between specific voxel SUVR_WM_ values and the probability of identifying Cog-LC. According to the beta coefficients in the representative PLS model, increased SUVR_WM_ in widespread brain regions was identified as a crucial contributor to distinguishing patients from HCs (Fig. 4C). These findings demonstrate that the PLS algorithm, based on the AMPAR density obtained from [^11^C]K-2 PET images, effectively differentiates patients with Cog-LC from HCs.

## Discussion

Using [^11^C]K-2 AMPAR PET imaging, our study successfully revealed a systemic increase in brain AMPAR density in patients with Cog-LC, showing that the increased AMPAR density was associated with cognitive impairment as assessed using RBANS scores, with the limitation that the race of the target population was restricted to Asians living in Japan. In this study, none of the participants had neurological comorbid or prior conditions known to affect cognitive function (Supplementary Table 1). Although some subjects did score on the MADRS, HAM-D and MDS-UPDRS, none met diagnostic criteria for mood or movement disorders. We also documented all concomitant medications at the time of [¹¹C]K-2 PET imaging (Supplementary Table 1). We have previously examined that major central nervous system medications did not affect [^11^C]K-2 PET image.^28^ Therefore, we considered most of the observed changes in cerebral AMPAR to Cog-LC.^28^ To our knowledge, this study is the first to suggest a potential relationship between synaptic functional molecules and cognitive impairment as a consequence of post-COVID-19 conditions. Previous imaging studies have focused on morphological,^11,12^ blood flow changes, cellular glucose metabolism^22,23^ and gliosis.^26^ Particularly in FDG-PET studies, astrocytic dysfunction has been suggested in Cog-LC, potentially impairing synaptic glutamate clearance and recycling.^24^ This disruption may disturb glutamate homeostasis leading to a pathological upregulation of post-synaptic AMPARs on the cell surface. [¹⁸F]FEPPA PET studies in Cog-LC have revealed persistent gliosis,^26^ indicating sustained neuroinflammation driven by elevated cytokines such as TNF-α. While our study did not directly measure gliosis, our findings are consistent with this mechanism: we observed that increased postsynaptic AMPAR density on the neuronal surface was positively correlated with plasma levels of TNFSF12, a member of the same TNF superfamily. Our study deepens the understanding of the molecular pathology of Cog-LC by revealing changes in glutamate AMPAR density in the living human brain.

The target population demonstrated a slight increase in depression, MADRS, and HAM-D scores (Table 1), suggesting that the global upregulation of AMPAR may not be exclusively attributable to cognitive impairment. However, depressive symptoms in this population did not meet the diagnostic criteria for major depressive disorder, as defined by the DSM-5 or ICD-10, and were instead subclinical symptoms secondary to cognitive impairment. Therefore, we hypothesized that the global upregulation of AMPAR is predominantly related to underlying cognitive impairment.

The Cog-LC cohort in our study attained significantly lower scores on the RBANS coding and figure recall tasks than the HCs. The RBANS coding score indicates executive function, attention and visual-motor coordination, while the figure recall score reflects visual memory. The LNS and MMSE scores of patients with Cog-LC were significantly lower than those of the HCs. The characteristics of Cog-LCs remain only partially established. However, recent studies have suggested that memory, attention and executive function are the core disabled domains of Cog-LC,^5^ which is consistent with our findings. However, it remains unclear whether the overall increase in AMPAR density across the brain also occurs in Cog-LCs when other cognitive functions are impaired. This is a limitation of this study.

These findings provide important insights. When the brain processes information, it needs to integrate various sensory and environmental inputs to function efficiently, which requires regulating brain activity to maintain an optimal state, avoiding hyperactivity and underactivity.^46^ Therefore, the increased synaptic AMPAR density in the entire brain in patients with Cog-LC might plausibly disrupt the signal-to-noise ratio or optimal brain stability in information processing, causing cognitive impairments. These are merely insights, and whether there is a causal relationship between elevated AMPAR density across the brain and cognitive dysfunction remains unclear. This is another limitation of this study because it primarily demonstrated the correlation between the increase in AMPAR density across the brain and cognitive decline. Another possible interpretation of the results is the imbalance of excitation to inhibition (E/I balance impairment). Since we did not conduct PET imaging with tracers for GABA receptors, it is the limitation of our study to estimate the E/I balance in this current study. Although electroencephalography (EEG)-based approaches have been proposed to estimate E/I balance, our study did not include EEG recordings, and thus such estimation could not be performed—this represents a methodological limitation. However, these approaches remain inferential and lack the molecular specificity afforded by techniques such as PET imaging.^47,48^

A vital implication of our findings is that suppression of AMPAR activity in the brain may be a viable treatment strategy for Cog-LC. We demonstrated that AMPAR density was significantly elevated across the entire brain in patients with Cog-LC compared to HCs (Fig. 2A and B). The systemic increase in AMPAR expression may result from compensatory mechanisms for the unknown loss of brain function. However, we found a significant negative correlation between RBANS subscale scores, Picture Naming and Figure Recall, and [^11^C]K-2 uptake across all brain regions, where [^11^C]K-2 uptake was higher in patients than in healthy participants (Fig. 2C–F). As we observed no significant negative correlation between these RBANS subscales and depressive scales (Supplementary Table 2), the cognitive impairments of Cog-LC were mediated not by depressive symptoms but by AMPARs upregulation after SARS-CoV-2 infection. These findings suggest that a widespread increase in AMPAR expression across the brain represents a pathological condition. Glutamate AMPARs are trafficked to post-synaptic membranes in an experience-dependent manner, where cell surface AMPARs form functional connections among neurons.^14^ The AMPARs on the cell surface are more critical components than intracellular AMPARs. Previous validations suggest that [¹¹C]K-2 PET imaging specifically indicates AMPARs on the cell surface.^30^ Therefore, the upregulated [¹¹C]K-2 SUVR_WM_ in patients with Cog-LC may indicate increased surface-expressed AMPAR density. Therefore, non-competitive antagonists of AMPAR, such as perampanel,^49^ may be a therapeutic candidate for Cog-LC. This should be tested in future randomized controlled trials.

Another interpretation of our findings is the potential utility of [^11^C]K-2 PET imaging for establishing a diagnostic framework for Cog-LC. Using a machine learning algorithm based on PLS, we distinguished the [^11^C]K-2 PET imaging of participants with Cog-LC and HCs with high sensitivity and specificity (Fig. 4B). Therefore, [^11^C]K-2 PET imaging holds promise as a robust diagnostic tool for Cog-LC, particularly in cases of challenging diagnoses and when patients may not receive adequate medical support despite experiencing symptoms.^10^ This should also be tested in future large-scale external cohorts.

To calculate *BP*_ND_, patients with Cog-LC underwent a 60-min dynamic [^11^C]K-2 PET scan, as did healthy individuals. However, for clinical applications, a surrogate marker for *BP*_ND_ is necessary to reduce the burden on patients who undergo [^11^C]K-2 PET scans and to accelerate large-scale data collection across multiple sites. The surrogate marker should be obtained from a static scan, which requires less scan time than a dynamic scan does. We have previously demonstrated that SUVR_WM_ 30–50 min after [^11^C]K-2 injection is an appropriate surrogate marker of *BP*_ND_.^24,27,28^ Therefore, we constructed [^11^C]K-2 SUVR_WM_ images of patients with Cog-LC.

In the present study, we analysed the data under the assumption that the kinetics of [¹¹C]K-2 are consistent across groups, as supported by the sufficiently similar linearity observed in the LGA from 20 min post-injection in both groups. In addition, the shapes of tTAC were similar in both groups.^28^

The AMPAR PET tracer used in this study is labelled with ^11^C, with a short life of approximately 20 min. Consequently, its use is limited to facilities equipped with an on-site cyclotron. To overcome these constraints, we have successfully synthesized [^18^F]K-40,^50^ an ^18^F labelled tracer with a longer half-life, which exhibits comparable performance in probing AMPAR as [^11^C]K-2. Notably, [^18^F]K-40 enables the acquisition of SUVR images using WM as a reference region, comparable to [^11^C]K-2.^50^ Moving forward, we also plan to conduct large-scale studies using [¹⁸F]K-40,^50^ which can be widely distributed and utilized at various institutions, to establish AMPA-PET as a diagnostic tool for Cog-LC.

The fact that no previous animal experiments can confirm the results of our present study also represents a limitation of this study. Future studies should uncover why AMPAR expression increases, resulting in cognitive impairment, following SARS-CoV-2 infection. Our previous validation indicated that [¹¹C]K-2 PET imaging depicts cell surface AMPARs.^30^ therefore, this systemic increase in AMPAR presentation across the whole brain may result from enhanced trafficking of AMPARs to the cell surface and reduced endocytosis. Among the various immunological abnormalities in Long COVID,^51^ our study revealed that two key factors, CCL2 and TNFSF12, are significantly correlated with AMPAR expression. Aberration of immunological responses in Cog-LCs may induce the upregulated trafficking of AMPARs to cell surfaces across the brain, inducing cognitive impairments via the misprocessing of information. Recent studies have shown that TNF-α released from astrocytes has been reported to promote the trafficking of AMPA receptors to the neuronal cell surface, thereby enhancing excitatory synaptic transmission.^27^ In line with this mechanism, we observed a significant positive correlation between plasma TNF-α levels and the index of AMPAR density (SUVR_WM_) in the current study. Although TNFSF12 also belongs to the TNF superfamily, there is currently no direct evidence linking TNFSF12 to AMPAR trafficking. Future studies should elucidate how these factors enter the brain tissues through the blood–brain barrier and pathologically regulate AMPAR dynamics. We hypothesized that these factors disrupt AMPAR trafficking to the cell surface or impair endocytosis. To clarify how these factors upregulate AMPAR cell surface expression, reverse translational studies using animal models are essential to gain deeper insights into their underlying pathways. Future clinical and biological studies should elucidate similar post-viral cognitive impairments^52^ and myalgic encephalomyelitis/chronic fatigue syndrome^53^ to deepen our understanding of the interactions between the immune and nervous systems.

## Supplementary Material

fcaf337_Supplementary_Data

## Data Availability

All requests for raw and analysed data were promptly reviewed by the Yokohama City University Research Promotion Department to determine whether the request was subject to any intellectual property or confidentiality obligations and further inspected by the Yokohama City University Certified Institutional Review Board. Based on these approaches, the derived data will be released via a material transfer agreement from the corresponding author. Our Scripts for PLS analysis, and figure generation are openly available at https://github.com/stsugawa/AMPA_COVID_PLS.
