# Supplementary material for: Systemic increase of AMPA receptors associated with cognitive impairment of long COVID
Source: Brain Commun. 2025 Oct 1;7(5):fcaf337. doi: 10.1093/braincomms/fcaf337 (PMC12483584; doi:10.1093/braincomms/fcaf337)
Supplement: fcaf337_Supplementary_Data [file fcaf337_supplementary_data.docx]

**Supplementary Materials**

**Supplementary Data Tables**

**Supplementary Table 1. Demographic and clinical characteristics of cognitive impairments in Long COVID (Cog-LC)**

| Patient ID | Age | Sex | BMI, kg/m^2^ | Educational background | Number of vaccine doses prior to infection | Time of infection relative to Cog-LC | Number of infection episodes prior to PET imaging | Timing of PET imaging | Interval between infection and PET imaging (months) | MADRS score | HAM-D score | Neurological comorbidities/ past histories | Medication |
| --- | --- | --- | --- | --- | --- | --- | --- | --- | --- | --- | --- | --- | --- |
| 1 | 46 | M | 19.6 | Bachelor | 0 | Aug-21 | 1 | Nov-23 | 27 | 6 | 4 | (None) | Mecobalamin, Kampo |
| 2 | 36 | F | 24.1 | Associate degree | 3 | Jul-22 | 1 | Nov-23 | 16 | 11 | 6 | (None) | Alprazolam |
| 3 | 43 | M | 21.4 | Bachelor | 3 | Aug-22 | 1 | Dec-23 | 16 | 2 | 0 | (None) | (None) |
| 4 | 45 | F | 32.9 | Associate degree | 4 | Oct-22 | 1 | Dec-23 | 14 | 23 | 13 | (None) | Eszopiclone, Lemborexant, Sodium Ferrous Citrate, Polaprezinc, vilanterol trifenatate/fluticasone furoate, Pitavastatin, Azilsartan/Amlodipine Besilate, Kampo |
| 5 | 40 | F | 26.2 | Bachelor | 2 | Dec-22 | 1 | Jan-24 | 13 | 11 | 6 | (None) | (None) |
| 6 | 39 | M | 26.1 | Associate degree | 2 | Feb-22 | 1 | Jan-24 | 23 | 4 | 6 | (None) | (None) |
| 7 | 28 | M | 21.5 | Bachelor | 0 | Sep-22 | 2 | Jan-24 | 16 | 3 | 2 | (None) | (None) |
| 8 | 39 | F | 20.6 | Bachelor | 2 | Oct-22 | 1 | Jan-24 | 15 | 15 | 9 | Migraine | Diazepam, Eperisone, Tizanidine hydrochloride |
| 9 | 35 | F | 24.4 | Bachelor | 0 | Mar-21 | 2 | Feb-24 | 35 | 20 | 21 | (None) | Clostridium butyricum combined drug, Esomeprazole, Sennoside, Fexofenadine hydrochloride, Clotiazepam, Rupatadine fumarate, d-Chlorpheniramine maleate, Celecoxib, Epinastine hydrochloride |
| 10 | 45 | F | 25 | High-school graduate | 0 | Aug-22 | 1 | Feb-24 | 18 | 15 | 14 | (None) | (None) |
| 11 | 46 | F | 23.8 | Bachelor | 3 | Aug-22 | 1 | Feb-24 | 18 | 8 | 6 | (None) | Hydrocortisone, Magnesium oxide, Clostridium butyricum drug, Glutathion |
| 12 | 23 | F | 21.7 | Bachelor | 1 | Jul-23 | 1 | Feb-24 | 7 | 2 | 4 | Hypothyroidism | Levothyroxine sodium hydrate, Ibudilast, Benidipine hydrochloride, Nicorandil, Isosorbide dinitrate |
| 13 | 39 | M | 24.1 | Master or more | 0 | Apr-20 | 1 | Mar-24 | 47 | 12 | 7 | (None) | Sertraline hydrochloride, Zolpidem tartrate, Adenosine triphosphate disodium hydrate, Ramelteon, Lemborexant, Polycarbophil calcium, Eperisone hydrochloride, Ibudilast, Clostridium butyricum combined drug |
| 14 | 49 | M | 24.3 | College non-completer | 2 | Jan-24 | 2 | Mar-24 | 19 | 7 | 6 | Meningitis (6-year-old) | Kampo |
| 15 | 43 | M | 28.6 | Bachelor | 0 | Jan-21 | 2 | Mar-24 | 38 | 9 | 8 | (None) | Ketotifen fumarate, Montelukast sodium, Ramosetron hydrochloride, Naproxen, Diphenhydramine salicylate/Diprophylline, Lemborexant, Kampo |
| 16 | 57 | M | 27.5 | Master or more | 3 | Mar-20 | 3 | Apr-24 | 49 | 10 | 5 | (None) | Amlodipine besilate, Pitavastatin calcium hydrate, Vonoprazan fumarate, Pemafibrate, Prosultiamine, Tocopherol nicotinate |
| 17 | 56 | M | 25.3 | Foundation degree | 5 | Sep-23 | 1 | Jun-24 | 9 | 11 | 13 | (None) | Polaprezinc, Kampo |
| 18 | 52 | F | 23.3 | Associate degree | 2 | Aug-22 | 1 | Jun-24 | 22 | 1 | 7 | (None) | Polaprezinc, Clostridium butyricum combined drug, Kampo |
| 19 | 27 | M | 20.9 | Master or more | 3 | Jun-22 | 1 | Jun-24 | 24 | 5 | 6 | (None) | Duloxetine hydrochloride, Lemborexant, Mosapride citrate hydrate |
| 20 | 34 | F | 15.7 | Master or more | 4 | Dec-22 | 1 | Jun-24 | 18 | 5 | 12 | (None) | Ketotifen fumarate, Fluvoxamine maleate, Diphenhydramine salicylate/Diprophylline, Dienogest, Ifenprodil tartrate, Clotiazepam, Kampo |
| 21 | 44 | F | 28.7 | Bachelor | 2 | Apr-22 | 1 | Jul-24 | 27 | 10 | 8 | (None) | Polaprezinc, Famotidine, Clostridium butyricum combined drug, Neurotropin, Lemborexant, Sodium valproate, Sodium ferrous citrate, Ascorbic acid/Calcium pantothenate, Kampo |
| 22 | 40 | F | 21.1 | High-school graduate | 3 | Feb-22 | 1 | Jul-24 | 29 | 12 | 11 | Febrile seizure (2-year-old) | Pyridostigmine bromide, Clostridium butyricum drug, Bifidobacterium, Magnesium oxide |
| 23 | 45 | F | 20.6 | Bachelor | 3 | Aug-23 | 1 | Jul-24 | 11 | 21 | 16 | Premenstrual syndrome | Norethisterone, Ursodeoxycholic acid |
| 24 | 27 | M | 19.2 | Bachelor | 3 | Aug-23 | 2 | Jul-24 | 11 | 5 | 12 | (None) | Sertraline hydrochloride, Fursultiamine hydrochloride, Kampo |
| 25 | 43 | M | 24.8 | Bachelor | 3 | Aug-22 | 1 | Jul-24 | 23 | 15 | 12 | (None) | L-Glutamine, Famotidine, Pemafibrate, Rosuvastatin calcium, Kampo |
| 26 | 47 | F | 23.3 | Associate degree | 0 | May-23 | 1 | Jul-24 | 14 | 5 | 3 | (None) | Drospirenone/Ethinylestradiol betadex |
| 27 | 24 | F | 20.6 | Bachelor | 2 | Mar-24 | 2 | Aug-24 | 5 | 8 | 7 | (None) | Hydrocortisone, Ibudilast, Ubidecarenone, Clostridium butyricum drug, Cetotiamine hydrochloride hydrate, Taurine, Kampo |
| 28 | 54 | M | 26.5 | Bachelor | 0 | May-21 | 1 | Aug-24 | 39 | 8 | 1 | (None) | Doxycycline hydrochloride hydrate, Ursodeoxycholic acid, Bepotastine besilate, Febuxostat, Hydroxyzine pamoate, Loxoprofen sodium hydrate |
| 29 | 48 | F | 36.8 | Associate degree | 0 | Apr-20 | 1 | Aug-24 | 52 | 13 | 7 | (None) | Nifedipine, Candesartan cilexetil, Loxoprofen sodium hydrate, Rebamipide, Relugolix |
| 30 | 45 | F | 20 | College non-completer | 1 | May-23 | 1 | Aug-24 | 15 | 20 | 13 | (None) | Etizolam, Clostridium butyricum combined drug, Albumin tannate, Kampo |

M, Male; F, Female; BMI, body mass index; MADRS, Montgomery Asberg Depression Rating Scale; HAM-D, 21-item Hamilton Depression Rating Scale

**Supplementary Table 2. Correlation between depressive scales and RBANS Picture Naming, Figure Recall**

|  | HAM-D vs. Picture Naming | HAM-D vs. Figure Recall | MADRS vs. Picture Naming | MADRS vs. Figure Recall |
| --- | --- | --- | --- | --- |
| Pearson r |  |  |  |  |
| r | -0.05817 | 0.07014 | 0.09603 | 0.2705 |
| 95% confidence interval | -0.4098 to 0.3086 | -0.2976 to 0.4198 | -0.2737 to 0.4410 | -0.09945 to 0.5748 |
| R squared | 0.003383 | 0.00492 | 0.009221 | 0.07318 |
| P value |  |  |  |  |
| P (two-tailed) | 0.7601 | 0.7126 | 0.6137 | 0.1482 |
| P value summary | ns | ns | ns | ns |

HAM-D, 21-item Hamilton Depression Rating Scale; MADRS, Montgomery Asberg Depression Rating Scale; ns, not significant.

**
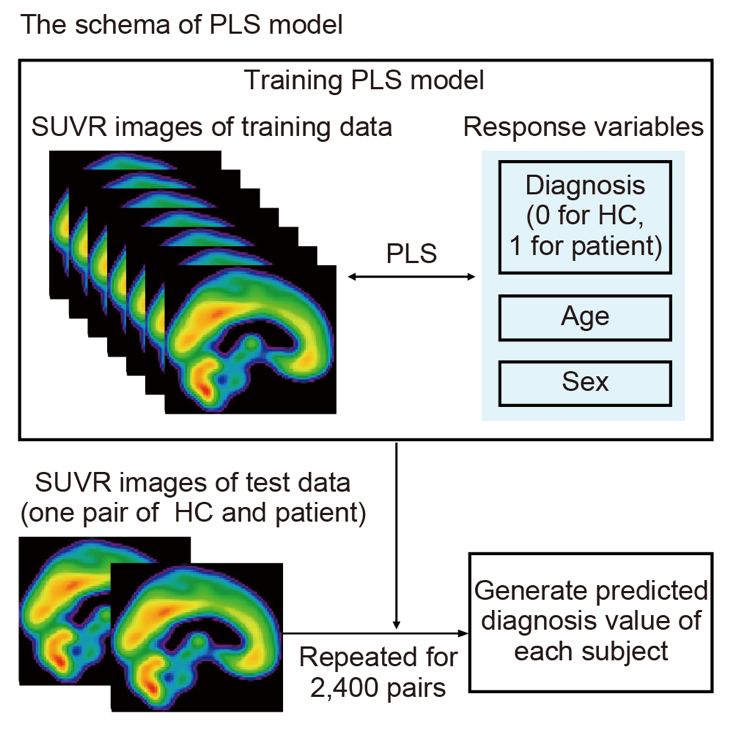
Supplementary Figures**

**Supplementary Fig. 1**. **Schema of the partial least squares (PLS) algorithm for distinguishing Cog-LCs from HCs.**

We employed a PLS algorithm using SUVR values as explanatory variables and disease status (0 for HCs and 1 for Cog-LCs), along with age, and sex as the response variables. Model performance was assessed using a leave-one-pair-out cross-validation approach. The mean predicted value for each participant was calculated by averaging their respective predicted values.

**
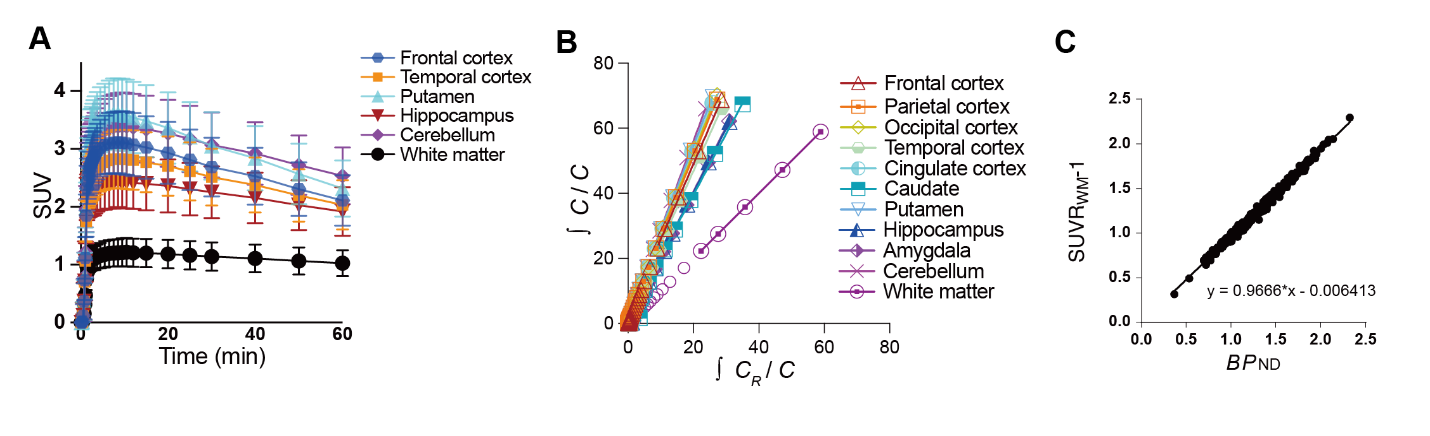
**

**Supplementary Fig. 2**. **Quantitative analysis of [^11^C]K-2 binding kinetics in cognitive impairments in Long COVID (Cog-LC)**

**A,** Averaged tissue time-activity curves (tTACs) in brain regions of patients with Cog-LC (*n* = 28, Two of thirty participants were omitted as only 30-50 min data were acquired). Each point and bar represent mean ± SD. **B,** Logan graphical analysis (LGA), where the ratio between an integrated tTAC in the reference region (white matter; *C*_R_) and a tTAC (*C*), and an integrated *C* and *C*, are plotted on the x- and y-axes, respectively. LGA of a representative Cog-LC is presented. **C**, Correlation between SUVR_WM_-1 and *BP*_ND_ obtained from LGA in 10 brain regions in patients with Cog-LC (Pearson correlation analysis, correlation coefficient = 0.9932, *P* < 0.0001, *y* = 0.955x + 0.02278). Each point represents SUVR_WM_-1 and *BP*_ND_ of a brain region in a participant.

Abbreviations: SUV, Standardized Uptake Value; SUVR, Standardized Uptake Value Ratio; *BP*_ND_, Binding Potential Non-Displaceable.

**
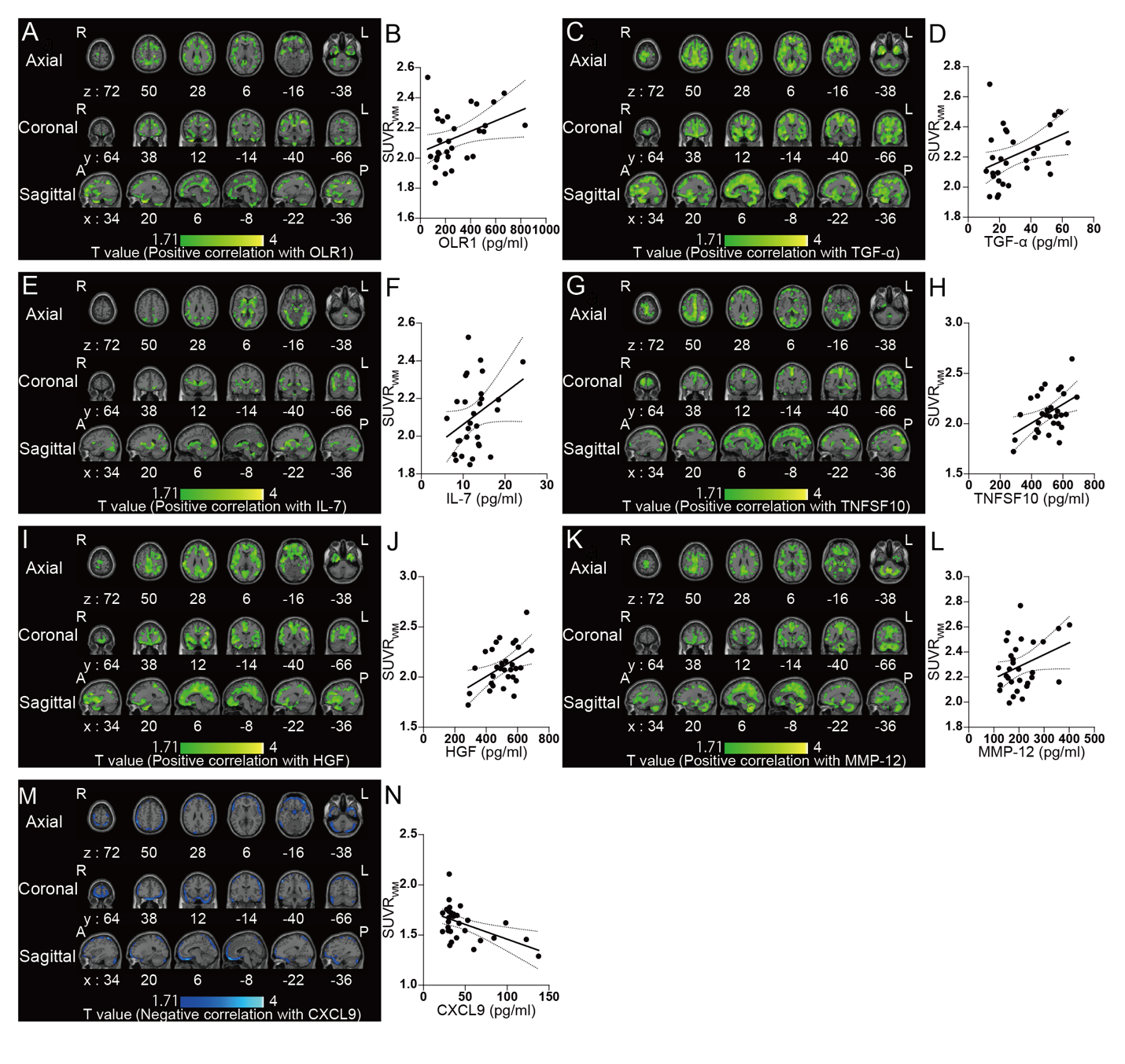
Supplementary Fig. 3**. **Correlation between AMPAR density and immunological factors in the plasma of patients with Cog-LC**

Brain regions showing a significant correlation between SUVR_WM_ and plasma OLR1 (**A**), TGF-α (**C**), IL-7 (**E**), TNFSF10 (**G**), HGF (**I**), MMP-12 (**K**), and CXCL9 (**M**) concentrations in patients with Cog-LC (n = 30) (*P* < 0.05, positive correlation; T > 1.71, negative correlation; T < 1.71, one-tailed, FDRc ). Correlation between averaged SUVR_WM_ in significant cluster and plasma concentrations of OLR1 (**B**, two-tailed Pearson correlation analysis, correlation coefficient = 0.3819, **P* = 0.0373), TGF-α (**D**, correlation coefficient = 0.3956, **P* = 0.0305), IL-7 (**F**, correlation coefficient = 0.3356, **P* = 0.0699), TNFSF10 (**H**, correlation coefficient = 0.4587, **P* = 0.0108), HGF (**J**, correlation coefficient = 0.2716, **P* = 0.1466), MMP-12 **(L**, correlation coefficient = 0.3398, **P* = 0.0662), and CXCL9 (**N**, correlation coefficient = -0.5012, **P* = 0.0048). Each point represents each participant.

Abbreviations: OLR1, oxidized low density lipoprotein receptor 1; TGF-α, transforming growth factor alpha; IL-7, interleukin-7; TNFSF10, tumour necrosis factor superfamily 10; HGF, hepatocyte growth factor; MMP-12, matrix metallopeptidase 12; CXCL9, C-X-C motif chemokine ligand 9; SUVR_WM_, Standardized Uptake Value Ratio using white matter region as reference; A, anterior; P, posterior; R, right; L, left; FDRc, false discovery rate correction

**Supplementary Methods**

**1. [^11^C]K-2 PET Images of Healthy Individuals**

For comparison with HCs, this Cog-LC study predefined data transfer from a healthy participant study (jRCTs031200083), which aimed to evaluate AMPAR density in healthy individuals using [^11^C]K-2. This trial (jRCTs031200083) was conducted at Yokohama City University Hospital, Keio University Hospital, Kyushu University Hospital, and the University of Fukui Hospital between 25 September 2020 and 7 October 2023. All participants provided written informed consent after receiving detailed information regarding the research protocol.

***1-1 Exclusion Criteria of Participants***

Patients were excluded if they met any of the following criteria: (1) PCR or antigen test not confirming a SARS-CoV-2 infection history; (2) Pregnancy, breastfeeding, or planning of pregnancy during the study period; (3) Blood hCG level ≥ 6 mIU/mL, (4) Hypersensitive to alcohol; (5) Blood tests showing AST, ALT, or ALP levels ≥2.5 times the upper limit of normal or serum Cr ≥ 2.0 mg/dL; (6) Epilepsy diagnosis before or after SARS-CoV-2 infection; (7) Psychiatric disorder diagnosis before or after SARS-CoV-2 infection; (8) Diagnosis of neurological disorders before or after SARS-CoV-2 infection; (9) Diagnosis of substance-related disorders (excluding nicotine and caffeine) before or after SARS-CoV-2 infection; (10) Positive urine screening for dependent substances (excluding prescribed medications); (11) Requiring continued use of perampanel or topiramate; (12) Unsuitable for 3T MRI (e.g., non-MRI-compatible implanted metals, large tattoos, severe claustrophobia, or body size incompatible with MRI scanner); (13) Potential participation in other clinical trials involving unapproved treatments or examinations during the study; (14) The principal investigator or coinvestigators deeming the participant ineligible.

**1-2 In vivo PET Imaging and MRI**

Detailed PET imaging and MRI protocols were implemented across the participating medical centres. The specific imaging parameters for each site are described below.

*Yokohama City University Hospital.* PET scans were conducted at Yokohama City University Hospital using four systems: Toshiba Aquiduo (Toshiba Medical, Gunma, 324-8550, Japan), Celesteion PCA-9000A/2A (Canon Medical, Gunma, 324-8550, Japan), Discovery MI (GE Healthcare Japan), and Cartesion (Canon Medical, Gunma, 324-8550, Japan). The Aquiduo system offered an axial field of view (FOV) of 240 mm with 80 contiguous slices, each 2.0 mm thick. A 4.7-second Computed Tomography (CT) scan for attenuation correction (AC) was performed, followed by intravenous administration of [^11^C]K-2 over 60 seconds. Emission data were acquired for 60 min using different frames: 18 frames of 10 seconds, 2 frames of 30 seconds, 7 frames of 60 seconds, 1 frame of 2 minutes, 1 frame of 3 minutes, 3 frames of 5 minutes, and 3 frames of 10 minutes each. Dynamic images were reconstructed using a 2D-OSEM algorithm with four iterations, 14 subsets, a 128 × 128 matrix, a zoom factor of 2.8, and a 5.0-mm Gaussian filter. Celesteion provided a 240 mm axial FOV with 96 consecutive 2.0-mm thick slices. After a 15.2-second CT, [^11^C]K-2 was intravenously injected for 60 seconds, followed by a 60-minute emission scan in 35 frames. Dynamic images were reconstructed using TOF 3D-OSEM with 2 iterations, 20 subsets, 128 matrices, a zoom factor of 1.0, and a 5.0-mm Gaussian filter. Discovery MI provided a 256 mm axial FOV with 89 consecutive 2.8-mm thick slices. After a 2.83-s CT scan, [^11^C]K-2 was intravenously injected for 60 seconds, followed by a 60-minute emission scan in 35 frames. Dynamic images were reconstructed using TOF 3D-OSEM with 2 iterations, 17 subsets, 256 matrices, and a 4.0-mm Gaussian filter. The Cartesion provided a 253.64 mm axial FOV with 256 consecutive 1.05682 mm thick slices. After a 5-second CT scan, [^11^C]K-2 was intravenously injected for 60 seconds, followed by a 60-minute emission scan in 35 frames. Dynamic images were reconstructed using TOF 3D-OSEM with 6 iterations, 12 subsets, 240 matrices, and a 4.0 mm Gaussian filter. Similarly, every participant underwent an MRI using a GE DISCOVERY MR750 (General Electric Medical Systems). In addition, 3D T1-weighted anatomical images (3D-T1WI) were acquired with a voxel size of 0.9 × 0.9 × 0.9 mm, a repetition time (TR) of 7.016 ms, an echo time (TE) of 3.056 ms, a flip angle (FA) of 8°, and a FOV of 220 mm, with a 256 × 256 matrix.

*Kyushu University Hospital.* PET imaging was performed using Biograph Vision (Siemens Healthcare) and Biograph mCT scanner (Siemens Healthcare). Vision provided an axial FOV extended 357 mm, encompassing 263 contiguous slices with a thickness of 1.0 mm. The imaging protocol began with a 26.67-second CT, followed by a 60-second intravenous administration of [^11^C]K-2. Subsequently, a 60-minute emission scan was performed, comprising 35 frames. Image reconstruction was performed using 3D-OSEM techniques with 8 iterations, 5 subsets, and a 5.0-mm Gaussian filter. The mCT provided an axial FOV of 250 mm, encompassing 165 contiguous slices with a thickness of 1.0 mm. The imaging protocol began with a 9.38 s CT, followed by a 60-second intravenous administration of [^11^C]K-2. Subsequently, a 60-minute emission scan was performed, comprising 35 frames. Image reconstruction was conducted using 3D-OSEM techniques with 8 iterations, 21 subsets, and a 5.0-mm Gaussian filter. Each participant underwent an MRI using the 3D MPRAGE protocol on an Ingenia 3.0 T scanner (Phillips). The 3D-T1WI acquisition parameters included voxel dimensions of 1.2 × 1.0 × 1.0 mm, TR/TE values of 6.8/3.1 ms, a 9° FA, 170 mm FOV, and a 256 × 256 matrix.

*University of Fukui Hospital.* During the PET scan, a 60-second intravenous administration of [^11^C]K-2 was performed, followed by a 60-minute emission scan of 35 frames. The 3D radial MR acquisition for the zero-echo time (ZTE) method^1^ in the axial direction was performed for AC of PET data with the following parameters: FOV 256 mm, 89 contiguous 2.78-mm thick slices, bandwidth ± 62.5 kHz, and an acquisition duration of 41 seconds. An MR-AC map was generated using the ZTE-AC technique. Image reconstruction was performed using 3D-OSEM + TOF with 3 iterations and 28 subsets, employing a 128 matrix and a 5.0-mm Gaussian filter. High-resolution 3D-T1WI acquisition parameters included voxel dimensions of 0.9 × 0.9 × 0.9 mm, TR/TE values of 8.5/3.3 ms, an 8° FA, 196 mm FOV, and a 256 × 256 matrix.

**2. Tissue Time-Activity Curve (tTAC) Acquisition**

For each participant, PET and 3D T1-weighted anatomical images (T1WI) were subjected to spatial normalisation to the Montreal Neurological Institute (MNI) standard space with Hammers’ atlas.^2^ This process was executed using the PNEURO tool (version 3.807, PMOD Technologies). To establish a reference region, we defined the VOI for WM using SPM 8 software, as previously described.^3^ Subsequently, TACs were generated for the defined brain regions using these VOIs. To quantify tracer uptake, we calculated the standardised uptake value (SUV) for each brain region. The SUV was computed as the ratio of the radioactivity concentration in the region at time t (Bq/mL) to the injected dose (Bq) divided by body weight (kg). This standardisation enabled the comparison of tracer uptake across different brain regions and between participants.

**3. Plasma Sampling and Proteomics**

To investigate which immunological factors are associated with increased cell surface AMPARs in patients with Cog-LC, we collected plasma samples from 30 patients with Cog-LC who underwent [^11^C]K-2 PET scans. Proteomics of plasma samples from patients with Cog-LC was conducted by combining a proximity extension assay with next-generation sequencing (Olink; Olink Proteomics AB, Uppsala, Sweden),^4^ which enables the examination of the concentrations of 45 immunological factors in plasma samples.

Plasma samples from patients with Cog-LC were analysed using the Olink® Target 48 Cytokine panel (Olink Proteomics AB, Uppsala, Sweden) based on the Proximity Extension Assay. In this method, pairs of oligonucleotide-labelled antibody probes bind to their target proteins, and when brought into proximity, the oligonucleotides hybridise. DNA polymerase addition triggers proximity-dependent DNA polymerisation, generating unique PCR target sequences. These sequences were detected and quantified using a microfluidic real-time PCR instrument. The data underwent rigorous quality control using internal extension controls and calibrators to adjust for intra- and interrun variations. Each sample plate was evaluated based on the standard deviation of the normalised protein expression values for the incubation and detection controls. The final results are expressed as absolute concentrations (pg/mL) using a 4-parameter logistic fit for quantification. Analytes with concentrations above or below the limit of detection in one of the samples were not analysed. Among the 45 inflammation-related protein biomarkers analysed, CCL8, IL-33, CXCL12, OLR1, IL-27, IL-2, CXCL9, TGF-α, IL-1β, IL-6, IL4, TNFSF12, TSLP, CCL11, HGF, FLT3LG, IL-17F, IL-7, IL-13, IL-18, CCL13, TNFSF10, CXCL10, IFN-γ, IL-10, CCL19, TNF, IL-15, CCL3, CXCL8, MMP-12, CSF2, CSF3, VEGFA, IL-17C, EGF, CCL2, IL-17A, OSM, CSF1, CCL4, CXCL11, LTA, CCL7, and MMP-1 were identified.

**Supplementary References**

1. Weiger M, Pruessmann KP. Short-T(2) MRI: Principles and recent advances. *Prog Nucl Magn Reson Spectrosc*. 2019;114-115:237-270.

2. Hammers A, Allom R, Koepp MJ*, et al.* Three-dimensional maximum probability atlas of the human brain, with particular reference to the temporal lobe. *Hum Brain Mapp*. 2003;19(4):224-247.

3. Miyazaki T, Nakajima W, Hatano M*, et al.* Visualization of AMPA receptors in living human brain with positron emission tomography. *Nat Med*. 2020;26(2):281-288.

4. Wik L, Nordberg N, Broberg J*, et al.* Proximity Extension Assay in Combination with Next-Generation Sequencing for High-throughput Proteome-wide Analysis. *Mol Cell Proteomics*. 2021;20:100168.
